# A Large Mass Displaying a Malignant Character on the Forearm: A Rare Case of Upper Extremity Pilomatrixoma

**DOI:** 10.7759/cureus.44728

**Published:** 2023-09-05

**Authors:** Burak Ozturk, Muhammed Yusuf Afacan, Ali Osman G Cibikci, Mahmut Kursat Ozsahin, Huseyin Botanlioglu

**Affiliations:** 1 Department of Orthopaedics and Traumatology, Istanbul University-Cerrahpasa, Cerrahpasa Medical Faculty, Istanbul, TUR

**Keywords:** tru-cut biopsy, ghost cell, large forearm mass, hair follicle benign tumor, marginal resection, pilomatrixoma

## Abstract

In this study, a 50-year-old male patient had a painless swelling on his right forearm. The lump on the forearm started one year ago and increased in size in the last two months. The mass was 3x6 cm and had a malignant appearance on radiological imaging. The case was reported as pilomatrixoma in the histopathological examination after marginal excision. In this case report, we emphasized that pilomatrixoma is one of the diagnoses we considered in mass formations that can be seen in the upper extremity, although rare. The large mass displaying a malignant character in radiological imaging can be pilomatrixoma, and the Tru-cut biopsy before the final surgery may help diagnosis by preventing the surgeons from aggressive surgical treatment. The marginal excision shall be enough in the definitive treatment. With this study, we aimed to discuss the place of pilomatrixoma in the orthopedic literature, which is published chiefly by otolaryngology, pathology, and dermatology clinics and lacks in the orthopedic literature because it rarely involves the extremities.

## Introduction

Pilomatrixoma is a rare skin tumor observed from pluripotent precursor matrix cells of the hair follicle. It is also known as the calcified epithelioma of Malherbe. In 2018, in a literature review, the cases were common in the first and second decades [1,2]. It is usually in the head, neck, and upper extremities. It is a benign, nodular lesion under the skin and subcutaneous tissue and well-circumscribed. Patients are generally asymptomatic and visit the clinic because of swelling. Magnetic resonance imaging (MRI), computed tomography (CT), and superficial tissue ultrasonography can help in the diagnosis. A definitive diagnosis is histopathological. Surgical removal of the lesion is essential for treatment [3]. In this case report, we highlighted that pilomatrixoma is one of the diagnoses to consider in mass formations that can be detected in the upper extremities while being uncommon. With this analysis, we sought to address the absence of pilomatrixoma from the orthopedic literature because it mostly appears in publications from dermatology, pathology, and otolaryngology clinics and seldom affects the extremities.

## Case presentation

**Figure 1:**
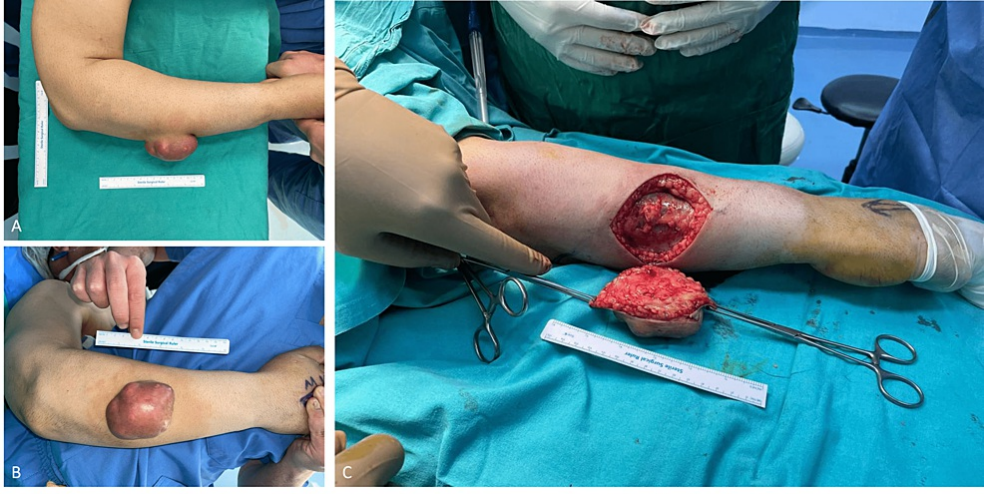
Preoperative and perioperative views of the mass. A: Lateral view of the mass before the operation. B: Anteroposterior view of the mass before the operation. C: Perioperative view of the mass, which was located superficially and was not associated with any neurovascular structure.

A 50-year-old male visited our clinic with a painless swelling on the right forearm. He was an active cigarette smoker and had no known comorbidity. The mass started one year ago and increased in size in the last two months. In the physical examination, an atypical malignant nodular 3 x 6 cm lesion was in the lateral 1/3 of the right forearm, with a slightly hyperemic appearance (Figure 1). 

**Figure 2:**
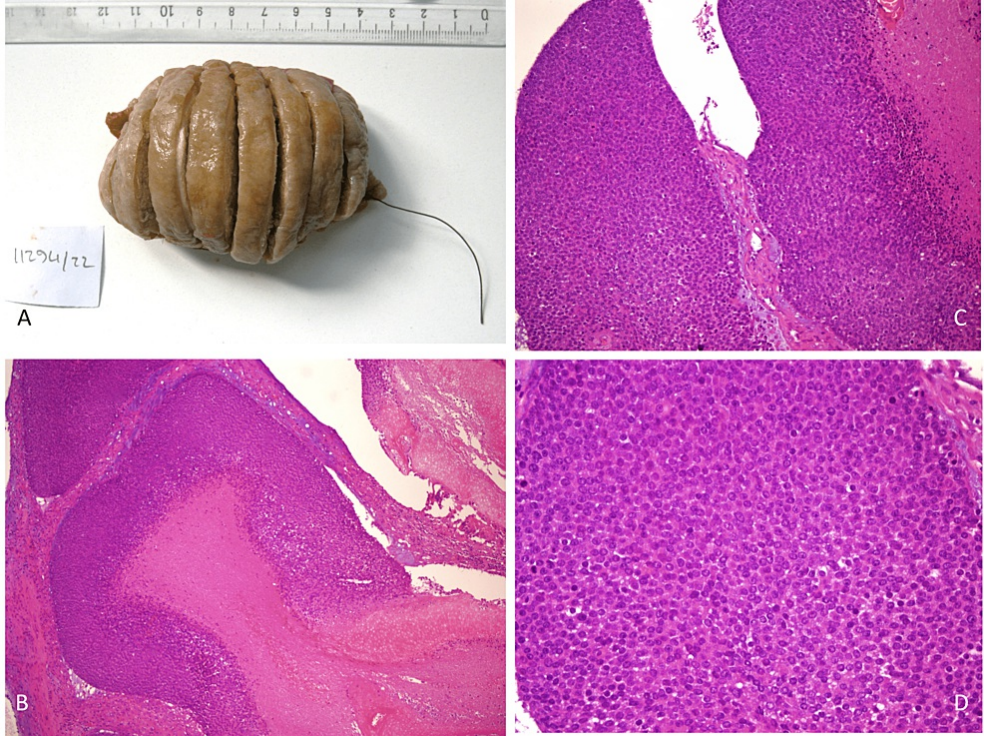
Macroscopic and microscopic views of the excised mass. A: Macroscopic appearance of the mass removed surgically as marginal resection. B: Microscopic view of the mass with Hematoxylin and Eosin stain (x40). A few dermal nodules extending to the hypodermis and monomorphic, small, borderless, basophilic cells with high mitotic activity in their periphery are seen in the specimen. These cells are keratinized towards the center of the nodules, with spaces appearing where the nuclei of these eosinophilic cytoplasmic cells were previously. These ghost cells appear toward the center of the nodules. As the lesion matures, the ratio of basophilic cells to ghost cells increases. The dermis surrounding the lesion is fibrotic, and granulomas are usually observed. C: Basophilic cells around the nodules and fibrotic dermis surrounding the lesion (Hematoxylin and Eosin stain x100). D: Basophilic cells in the periphery of the nodules (Hematoxylin and Eosin stain x100).

Neurovascular examination revealed no deficit. Ultrasonography revealed a soft tissue mass located subcutaneously. It was approximately 3x6 cm in size, and we observed peripheral point calcifications around the lesion. Contrast-enhanced MRI revealed a 44x28.5 mm mass formation (suspicion of sarcoma) with heterogeneous contrast enhancement, with a size of 44 x 28.5 mm in the subcutaneous fatty tissue of the forearm mid-section anterolaterally, after intravenous contrast material injection. There was no invasion of the bone tissue. F18-fluorodeoxyglucose positron emission tomography/computed tomography (FDG-PET/CT) examination revealed a soft tissue density mass, hypermetabolic soft tissue tumor (malignant sarcoma suspicion), located in the lateral of the right forearm middle part, located in the subcutaneous tissue, with intense FDG uptake (maximum standardized uptake value: 20.4) whose widest axial diameter measuring 3.0x5.8 cm. Ultrasonography-guided Tru-cut biopsy was applied to the patient by interventional radiology. The epithelial tumor with pilomatrical differentiation was pilomatrixoma. The patient underwent surgical marginal resection. After surgical marginal resection, the wound layers were sutured in accordance with their anatomy. The histopathological diagnosis of the excised material was pilomatrixoma (Figure 2). 

The patient had no active complaints in the postoperative follow-ups (first week, second week, first month, sixth week, second month, sixth month, first year), and there was no recurrence.

## Discussion

Pilomatrixoma is usually seen below 20 years of age. It is more common in females than males (F: M, 3/2) [4]. There are also rare cases seen in older age. Our case is a 50-year-old male patient. Although rare, there is also a malignant form. The malignant form is mostly seen in middle age and the last years of life. Malignant ones are known as 'Pilomatrix carcinoma'. Approximately 130 malignant cases known in the literature have been reported [5]. Since our patient was 50 years old, this differs from the literature review in terms of being older and benign. Pilomatrixoma is frequently seen in the head and neck (70%) region. It is seen less frequently in the upper extremities (22%) [6]. In our case, it was located in the upper extremity (right forearm). It is compatible with the literature and is in a rare localization. It usually presents as a solitary lesion. Multiple lesions (2-3.5%) are mostly familial. It can accompany diseases such as myotonic dystrophy, Turner syndrome, Gardner syndrome, and Rubinstein-Taybi syndrome [7,8]. Our case was also observed as a solitary lesion, and our patient did not have any known additional disease. Calcified hematoma, infected sebaceous cysts, calcified lymphadenopathy, epidermal cysts, giant cell tumors, and metastatic calcification should be considered in the differential diagnosis of pilomatrixoma [9]. The reason for the majority of patients to visit the clinic is painless, palpable swelling. Clinically, it appears as a mobile, painless, and blue-red mass. The dimensions of the mass can vary between 0.5 cm and 3 cm. The largest pilomatrixoma documented in the literature is 18 cm [10]. Pilomatrixomas larger than 7 cm are considered 'giant pilomatrixoma' [11]. Our case is clinically compatible with the literature and is larger than the commonly encountered pilomatrixoma. Radiological imaging helps in making the diagnosis. In ultrasonography, increased echogenicity appears as a subcutaneous mass with peripheral scattered punctate foci due to calcification, giving the impression of a peripheral hypoechoic halo [3]. It is reported that the incidence of calcification is 69%-85% [12]. As a result of the ultrasonographic examination of our patient, there were peripheral point calcifications around the soft tissue lesion. The definitive diagnosis of pilomatrixoma is made histopathologically. On histopathological examination, shadow (ghost) cells, basaloid cells, and calcification can be seen [13]. In the histopathological examination of the excised mass in our patient, the cells were keratinized toward the center of the dermal nodules, the appearance of eosinophilic cytoplasm became prominent, and ghost cells were formed in the area where the nucleus was previously. Surgical removal of the lesion is recommended for treatment [14]. Our patient underwent surgical marginal resection. There was no vascular, nerve, or bone invasion of the mass during the surgical operation. Although local recurrence is rare (2-6%), it is associated with inadequate resection [15]. No recurrence was observed in our patient's one-year follow-up. In the literature, it is not clear how long the patient should be followed up in terms of recurrence. Additional studies are needed for this.

## Conclusions

In this case report, we emphasized that pilomatrixoma is one of the diagnoses to consider in mass formations in the upper extremity despite being rare. Its treatment is surgical marginal excision of the mass and then close follow-up of the patient for recurrence. Although radiological and nuclear medicine imaging methods showed that the lesion had a malignant appearance in a patient who presented with a large mass with a malignant appearance on the forearm, the final pathology result may be pilomatrixoma. The preoperative Tru-cut biopsy shall guide the diagnosis, and marginal resection rather than aggressive surgery may also be sufficient as the definite treatment.
